# Ventilator-associated Pneumonia caused by commensal oropharyngeal a retrospective Analysis of a prospectively collected Database

**DOI:** 10.1186/s12890-015-0087-y

**Published:** 2015-08-12

**Authors:** Johannes B. J. Scholte, Johan I. M. van der Velde, Catharina F. M. Linssen, Helke A. van Dessel, Dennis C. J. J. Bergmans, Paul H. M. Savelkoul, Paul M. H. J. Roekaerts, Walther N. K. A. van Mook

**Affiliations:** Department of Intensive Care Medicine, Luzerner Kantonspital, 6000 Luzern 16, Switzerland; Department of Intensive Care Medicine, Maastricht University Medical Centre+, P.O. box 5800, 6202 AZ Maastricht, The Netherlands; Department of Medical Microbiology, Atrium Medical Centre, P.O. box 4446, 6401 CX Heerlen, The Netherlands; Department of Medical Microbiology, Maastricht University Medical Centre+, P.O. box 5800, 6202 AZ Maastricht, The Netherlands

**Keywords:** Ventilator-associated pneumonia, Bronchoalveolar lavage, Commensal oropharyngeal flora, Intensive care unit, MALDI-TOF-MS

## Abstract

**Background:**

The significance of commensal oropharyngeal flora (COF) as a potential cause of ventilator-associated pneumonia (VAP) is scarcely investigated and consequently unknown. Therefore, the aim of this study was to explore whether COF may cause VAP.

**Methods:**

Retrospective clinical, microbiological and radiographic analysis of all prospectively collected suspected VAP cases in which bronchoalveolar lavage fluid exclusively yielded ≥ 10^4^ cfu/ml COF during a 9.5-year period. Characteristics of 899 recent intensive care unit (ICU) admissions were used as a reference population.

**Results:**

Out of the prospectively collected database containing 159 VAP cases, 23 patients were included. In these patients, VAP developed after a median of 8 days of mechanical ventilation. The patients faced a prolonged total ICU length of stay (35 days [*P* < .001]), hospital length of stay (45 days [*P* = .001]), and a trend to higher mortality (39 % vs. 26 %, [*P* = .158]; standardized mortality ratio 1.26 vs. 0.77, [*P* = .137]) compared to the reference population. After clinical, microbiological and radiographic analysis, COF was the most likely cause of respiratory deterioration in 15 patients (9.4 % of all VAP cases) and a possible cause in 2 patients.

**Conclusion:**

Commensal oropharyngeal flora appears to be a potential cause of VAP in limited numbers of ICU patients as is probably associated with an increased length of stay in both ICU and hospital. As COF-VAP develops late in the course of ICU admission, it is possibly associated with the immunocompromised status of ICU patients.

## Background

Mechanically ventilated patients are at risk for ventilator-associated pneumonia (VAP) [1], which is associated with an increased intensive care unit (ICU) length of stay (LOS), morbidity and mortality [2–5]. A globally accepted gold standard for the diagnosis of VAP is lacking [6–8], with the exception of the new surveillance ventilator-associated events definitions [9]. Generally recommended and accepted microbiological diagnostic modalities for VAP are quantitative specimen collection techniques as bronchoalveolar lavage (BAL) [10, 11] and protected specimen brushing obtained bronchoscopically and nonbronchoscopically. The identification of aerobic oropharyngeal flora in BAL fluid (BALF) cultures is commonly considered colonization, contamination from the upper respiratory tract or otherwise regarded clinically irrelevant, regardless of quantity [11–13].

Authoritative guidelines remain inconclusive regarding the role of commensal oropharyngeal flora (COF) as a causative agent in VAP, mainly due to a scarcity of studies in this research field [14]. However, there is evidence that COF may cause pulmonary infection, mostly in immunocompromized patients. Examples are *Streptococcus oralis/mitis* in neutropenic patients [15, 16] and species of the *Streptococcus anginosus* group in patients with predisposing factors including cancer, alcoholism [17, 18], and cystic fibrosis [19, 20]. Furthermore, aspiration may cause a pneumonia due to both anaerobic and/or aerobic oropharyngeal flora [21]. For the critically ill, several studies demonstrate that the innate immune response is declined due to several different mechanisms [22–24]. Therefore, one may hypothesize that ICU patients are likewise at risk for infections by COF. This study explores whether COF can be a cause of VAP.

## Methods

### Setting

The study was conducted at the Maastricht University Medical Centre, a 715-bed hospital with approximately 30,000 annual admissions, 18 mixed surgical-medical ICU beds, and 9 post-cardiothoracic surgery beds. Other elective postoperative patients are rarely admitted, due to a 24-h post anaesthesia care unit. When technically possible and safe, a BAL was performed in all mechanically ventilated patients who met the clinical criteria of suspected VAP. These criteria include (≥2 of the following) a rectal temperature > 38.0 °C or < 35.5 °C, white blood cell count > 10,000/μl or < 3,000/μl, purulent sputum, and a new, persistent or progressive infiltrate on chest X-ray [25]. In patients with localized pulmonary lesions, the affected region was sampled, whereas in case of diffuse pulmonary lesions the middle lobe or lingula was lavaged. The BALF was microbiologically evaluated within 15 min after it was obtained. Selective oropharyngeal decontamination (SOD) is used since December 2010, whereas selective digestive tract decontamination (SDD) is used since January 2012. The SOD consists of topical antibiotics (polymyxin E, tobramycin, amphotericin B) applied to the oropharynx, whereas the SDD consists of oropharyngeal and gastric application of the same non-absorbable antibiotics along with a four day course of intravenous cefotaxime. The ethics committee of the institution, the “Medical Research Ethics Committee”, approved the study and informed consent was regarded unnecessary since standard care was provided.

### Definitions

Ventilator-associated pneumonia in clinically suspected cases (for definition, see previous paragraph) was diagnosed if subsequent BALF analysis was indicative for pneumonia: cultures yielding a potentially pathogenic microorganism [12] ≥ 10^4^ cfu/ml and/or if ≥ 2 % BALF cells containing intracellular organisms (ICOs) [10, 26]. In a pneumonia suspected case that was admitted from home less than 3 days prior to diagnosis, community-acquired pneumonia (CAP) was considered, if this case had no recent contact with the healthcare system. A CAP was also considered when the potentially pathogenic microorganism was very unlikely to be nosocomial (e.g. *Haemophilus influenzae*, *Mycoplasma* spp.) [27]. When a clinical suspected case was admitted in the hospital for more than 3 days and with positive BALF results, but was not mechanically ventilated for ≥ 48 in the 72 h prior to the pneumonia, hospital-acquired pneumonia (HAP) was diagnosed [14].

Commensal oropharyngeal flora as the cause of VAP was considered in VAP suspected cases if BALF quantitative cultures revealed COF ≥ 10^4^ cfu/ml without significant growth (≥10^4^ cfu/ml) of other potentially pathogenic microorganisms. Commensal oropharyngeal flora included (a combination of) the following bacteria: viridans streptococci, coagulase-negative staphylococci, *Haemophilus* spp. (excluding *H. influenzae* if not predominant), *Moraxella* spp. (if not predominant), *Corynebacterium* spp., *Neisseria* spp., *Peptostreptococcus* spp., *Stomatococcus* spp., and *Prevotella* spp. [28]. Whereas *Candida* spp. may occasionally cause a pneumonia [29], *Candida* spp. were considered nonpathogenic in this study, consistent with previous studies and guidelines [30–32].

### Data collection

From January 2005 until January 2014, all results of BALF analyses from patients consecutively admitted to the ICU were prospectively collected. From this database, patients with suspected COF as the cause for VAP were included in the present study. Cases lacking a microbiological BAL report were excluded. Retrospectively, the following clinical data were collected or calculated from the included cases: body temperature, C-reactive protein (CRP), white blood cell count, antibiotic administration, ICU length of stay, hospital length of stay, duration of mechanical ventilation, mortality, acute physiology and chronic health evaluation (APACHE)-II score (to calculate standardized mortality ratio [SMR; observed mortality divided by expected mortality]) [33], sequential organ failure assessment (SOFA) score (to determine the extent of critical illness) [34, 35], clinical pulmonary infection score (CPIS; more than 6 points is indicative of pneumonia) [36], and post-mortem examination, if available.

### Reference population

On the advice of the statistical department a reference population was used in order to place the results of suspected cases in perspective. Since June 2013, the hospital participated in the Dutch National Intensive Care Evaluation registry. To experience the possibilities of this registry, the characteristics of all patients admitted from June 2013 to April 2014 in the same ICU were extracted from this database. Post cardiothoracic surgery patients (44 % of all ICU admissions) were excluded. It should be realized that a reference population is not a control group and interpretation of finding should be performed in this perspective.

### Microbiological data collection

Bronchoalveolar lavage fluids were initially analysed according to a highly standardized protocol as described elsewhere [37]. From each BALF sample, 6 ml was centrifuged (250 g for 10 min), dividing the sample into cells and supernatant. The supernatant was stored in tubes of 1 ml at −80 °C. The cells were re-suspended in 6 ml of a mixture of Eagle’s Minimal Essential Medium with 2 % Dimethyl Sulfoxide and stored in tubes of 1 ml at −80 °C. Oropharyngeal flora was (formerly) reported on the basis of classical bacteriological phenotypic identification tests. In order to confirm and specify these results, included samples were defrosted and quantitatively cultured on blood agar, chocolate agar, and MacConkey agar. The different colony types were identified using matrix-assisted laser desorption/ionization time-of-flight mass spectrometry (MALDI-TOF-MS), a rapid and highly accurate soft ionization technique [38]. Antibiotic susceptibility was assessed for all separate strains when quantitative cultures revealed ≥ 10^4^ cfu/ml. Results of polymerase chain reactions (PCRs) for viruses and *Pneumocystis jirovecii*, as well as Grocott’s methenamine silver staining, were collected from electronic patient data organisers using SAP. In patients that were both admitted to the hospital less than 8 days prior to BAL procedure and that lacked PCR results, PCRs for the identification of respiratory viruses were performed on the defrosted BALF. These viruses included influenza virus A and B, human respiratory syncytial virus, human metapneumovirus and parainfluenza virus 1–4. Furthermore, results of endotracheal aspirates (ETA) were analysed, preferably from the day of BAL, otherwise one day before or after. Endotracheal aspirates were obtained twice weekly and in case of clinical suspicion of a pulmonary infection. Samples were immediately microbiologically evaluated when obtained during daytime.

### Commensal oropharyngeal flora caused ventilator-associated pneumonia likelihood

Based on the collected clinical, radiographic, and microbiological data and subsequent microbiological analyses made, the likelihood of presence of COF caused VAP (COF-VAP) was evaluated by 4 researchers (two consultant ICU physicians, one medical microbiologist, one ICU researcher).

### Statistics

Patient characteristics were analysed using descriptive statistics and presented as the mean ± standard deviation, median including interquartile range, or absolute numbers and percentages of patients, where applicable. Demographic and clinical characteristics of the suspected cases were compared with the reference population using the single sample *t-*test, the paired samples *t*-test or the Fisher’s exact test, where appropriate. For the comparison of the observed and expected mortality, a Chi-square test was used. A one-way within-subjects ANOVA was used to analyse the course of the SOFA-score, body temperature, leukocytes and CRP. Statistical significance was defined as *P* < .05. The IBM SPSS Statistics version 20 for Windows (Chicago, IL, USA) was used for analysis.

## Results

**Fig. 1:**
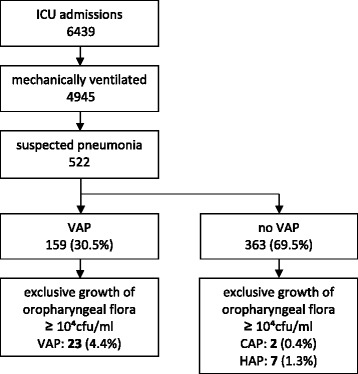
Inclusion flow chart

The study was conducted between January 2005 and July 2014. During this period, 17,254 patients were admitted to the ICU, of which 159 individuals met the VAP criteria during the study period. Twenty-three patients were considered to have COF-VAP (see Fig.  1 for inclusion flow chart). During the defined period, 899 patients (excluding post cardiothoracic surgery patients) needed ICU admission and consequently, these patients were included in the reference population.

Basic characteristics including clinical findings, BALF cytological results and outcome of the studied group and the reference population are presented in Table 1. Individual results of the analyses are presented in Table 2. The most common reason for ICU admission was respiratory failure (7/23 [30 %]) followed by multi-trauma (5/23 [22 %]). The reason for ICU admission of the study group was more often surgically related compared to the reference population (13/23 [57 %] *vs.* 267/899 [30 %], *P* = .01). Suspected COF-VAP developed after a median of 8 days of mechanical ventilation. Aspiration prior to BAL was considered possible in 3 patients. Five patients were mechanically ventilated for more than 2 days by means of a tracheostomy prior to the suspected VAP.

**Table 1:** Basic characteristics and clinical findings

	Commensal oropharyngeal flora VAP	Reference population	*P* value
	N = 23	N = 899	
Age, mean (SD)	55 (16)		
Male (%)	16 (70 %)	557 (62 %)	.52
Medical history (%)			
Cardiac	12 (52 %)		
Pulmonary	7 (30 %)		
Oncologic	7 (30 %)		
Immunocompromised	2 (9 %)	98 (11 %)	.85
Indication for ICU admission (%)			
Surgical	13 (57 %)	267 (30 %)	.01
Non-surgical	10 (43 %)	632 (70 %)	.01
ICU admission scores (SD) n = 21			
APACHE-II score	19.8 (9.9)	19.1 (8.4)	.75
Predicted mortality	31 % (26)	34 % (26)	.60
SOFA score	8.1 (3.3)		
Characteristics at the day of diagnosis (SD)			
SOFA score	6.9 (3.7)		
Body temperature (°C)	38.3 (1.1)		
WBC count (10^9^/L)	14.3 (8.4)		
CRP (mg/L)	180 (108)		
CPIS	6.4 (2.1)		
Median days on MV (IQR)	8 (4–19)		
Number of BAL fluid cells	6.9*10^5^ (1.7*10^5^)		
of which PMNs	60 % (35)		
of which containing ICOs	5.1 (6.5)		
Outcome			
ICU mortality	9 (39 %)	234 (26 %)	.16
SMR (SD)	1.26 (0.42)	0.77 (0.04)	.14
28-day mortality	3 (13 %)		
60-day mortality	6 (26 %)		
Median hospital LOS (IQR)	63 (50–146)	23.7 (=mean)	.001
Median ICU LOS (IQR)	33 (13–62)	5.7 (=mean)	.000
Median days on MV (IQR)	34 (17–67)		
Median hospital LOS after diagnosis (IQR)	22 (10–59)		
Median ICU LOS after diagnosis (IQR)	13 (7–55)		

If no number is provided, this information was not available in the Netherlands intensive care evaluation registry. APACHE-II score could not be calculated in 2 patients due to incomplete admission data

**Table 2:** Characteristics, diagnostic results and outcome in suspected cases of commensal oropharyngeal flora caused ventilator-associated pneumonia

Case	Sex	Admission indication	BAL fluid analysis			Endotracheal aspirate Semiquantitatively	Antibiotic treatment (days [0 = diagnose])	Additional characteristics	Diagnosis	ICU LOS
	Age		% ICOs	culture results in cfu/ml	MALDI-TOF-MS and re-culturing (cfu/ml)					
			% PMN							
1	M	Respiratory failure	0.0	COF 6*10^4^	No growth	COF few	Co-trimoxazole −7-27	Some COF possibly resistant to antibiotics	COF-VAP	92^a^
	60		11.6				Piperacillin −7-27			
2	F	Abdominal sepsis	0.0	COF 5*10^4^	Specimen storage lacking	*P. aeruginosa* heavy	Piper/tazob −4-11	Tracheostomy	COF-VAP or	31
	77		87.2	*P. aeruginosa* 2*10^3^, sensitive to piper/tazob		*C. albicans* few			*P. aeruginosa* VAP	
3	F	Abdominal sepsis	8.2	COF 2*10^5^	*R. dentocariosa* 2*10^2^	*S. aureus* heavy	Piper/tazob 0-2	Nocturnal CPAP for OSAS	COF-VAP	60
	57		85.6		*S. oralis* 7*10^2^		Vancomycin 2-9			
					*S. aureus* 6*10^2^					
4	F	Post cardiac arrest	10.8	COF 10^5^	*S. constellatus* 10^6^	*S. milleri* heavy	Piper/tazob 0-3	Possible aspiration	COF-VAP	33
	67		34.6	*S. milleri* 10^5^	*S. epidermidus* 3*10^3^		Penicillin 3-8			
5	M	Multi-trauma	2.5	COF 4*10^4^	*S. mitis/oralis* 5*10^3^	No growth	Amoxi/clav acid 1-8	Chronic obstructive pulmonary disease	COF-VAP	16
	47		91.5		*N. mucosa* 10^3^					
					*C. sputigena* 5*10^2^					
6	M	Neurological	4.8	COF 10^5^	*S. mitis/oralis* 5*10^3^	*Citrobacter* spp. few	Piper/tazob 0-6		COF-VAP	15
	23		68.2	*S. aureus* 10^3^	*S. aureus* 2*10^3^					
				*E. cloacae* 10^3^	*S. anginosus* 10^3^					
7	M	Multi-trauma	8.8	*P. melaninogenetica* 10^5^	Specimen storage lacking	COF few	Metronidazole 1-7	*P. melaninogenetica* not susceptible to Metronidazole	COF-VAP	9
	39		90.8							
8	M	Pneumococcal pneumonia	2.0	COF 10^5^	Specimen storage lacking	*P. aeruginosa* moderate	Ciprofloxacin 0-13	Tracheostomy. Pulmonary rehabilitation clinic.	COF-VAP	134
	64		21.2	*P. aeruginosa* 2*10^2^						
9	M	Respiratory failure	1.4	COF 3*10^4^	Specimen storage lacking	COF few	Gentamicin 0	Tracheostomy	COF-VAP	47^a^
	67		23.6				Ciprofloxacin −5-8	Post-mortem exam: severe chronic fibrotic inflammation and active pneumonia		
							Metronidazole −5-8			
							Vancomycin −5-8			
10	M	Respiratory failure	0.2	COF 10^5^	No growth	*C. albicans* rare	Ciprofloxacin −14-0	Lobectomy for aspergilloma. Imposible to wean from MV.	COF-VAP	70^a^
	62		99.6	*C. albicans* 10^5^			Piper/tazob −2-3			
							Fluconazole 3-17			
11	M	Cerebral hemorrhage	16	COF 10^4^	*L. acidophilus* 10^2^	*H. influenza* few	Piper/tazob 0-2		COF-VAP	11
	31		95.4	*P. melaninogenetica* 2*10^2^			Amoxi/clav acid 2-8			
12	M	Sepsis	0.0	COF >10^5^	*M. morganii* 2*10^3^	*M. morganii* moderate	Flucloxacilline −22- -3		COF-VAP	42^a^
	80		15.4		*S. malthophilia* 5*10^2^	*C. albicans* moderate	Rifampicine −17- -10			
						*K. pneumonia* few	Piper/tazob −3 -10			
							Co-trimoxazole 3-10			
13	M	Post cardiothoracic surgery	19.6	COF 3*10^4^	*S. anginosis* 10^3^	COF moderate	Piper/tazob 0-8			
		COF-VAP	63^a^							
	71		56.2		*S. constellatus* 10^3^	*C. freundii* moderate				
					*N. mucosa* 10^2^					
14	M	Multi-trauma	3.4	COF 10^5^	*E. faecalis* 6*10^2^	COF heavy	Piper/tazob 0-1	Tracheostomy	COF-VAP	62
	41		44.4	*P. aeruginosa* 7*10^3^	*S. mitis/oralis* 5*10^2^	*P. aeruginosa* heavy	Amoxi/clav acid 1-7			
15	M	Respiratory failure	NP	COF 10^4^	*E. faecalis* 2*10^4^	COF few	Co-trimoxazole −25 -13	Aids. ADV ct 17. CMV ct 37. *P. jirovecii* +*.* CT-thorax suggestive for PcP. Post-mortem exam: active pneumonia, possibly PcP, ADV and CMV.	PcP CAP, ADV CAP, and/or *E.faecalis* VAP	44^a^
	40		NP		*S. hominis* 2*10^2^		Piper/tazob 0-7			
							Vancomycin 0-8			
16	F	Respiratory failure	0.0	COF 10^5^	No growth	COF few	Piper/tazob −14- -4	Ileus. Post-mortem exam: faecal peritonitis. No pneumonia. Culture -	Abdominal sepsis	4^a^
	55		78.0	*C. glabrata* 4*10^2^		*C. albicans*	Ciprofloxacin −3-1			
				*C. albicans* 2*10^2^			Vancomycin −2-2			
17	F	Post abdominal surgery	0.0	COF 3*10^4^	Specimen storage lacking	COF few	Missing data		COF-VAP	13
	70		68.0							
18	M	Post neurological surgery	1.6	COF 2*10^4^	*S. aureus* 3*10^3^	*S. aureus* heavy	Piper/tazob ?-0		COF-VAP *or S. aureus* VAP	17
	58		94.4	*S. aureus* 3*10^3^, sensitive to piper/tazob			Flucloxacillin 0-10			
							Gentamicin 3-5			
19	M	Post-cardiothoracic surgery	0.0	COF 5*10^4^	Specimen storage lacking	COF few	Piper/tazob −8 - -1	Tracheostomy	COF-VAP	81
	73		2.8			*K. pneumoniae*	Meropenem −1-7			
20	M	Multi-trauma	19.4	COF 10^4^	*H. influenza* 10^6^	*H. influenza* heavy	Amoxi/clav acid −5- -4	Possible aspiration	*H. influenza* CAP	62
	49		95.0		*E. coli* 4*10^3^	OF few	Piper/tazob 0-5			
					*N. subflava* 4*10^3^	*S. aureus* few	Gentamicin 4			
21	F	Post cardiac arrest	0.0	COF 10^4^	*E. faecium* 5*10^3^	COF heavy	Piper/tazob 0-3	B- cel CLL. PIV-3 ct 19.	PIV-3 CAP or *E. faecium* VAP	22^a^
	45		1.0		*E. faecalis* 9*10^2^		Vancomycin 0-3	Post-mortem exam: active pneumonia. Cultures: CNS, *E. faecium* and *Candida* spp.		
					*S. epidermidis* 6*10^2^		Meropenem 2-3			
22	F	Cerebral hemorrhage	7.4	COF 10^4^	*C. koseri* 10^6^	COF heavy	Vancomycin 0-2	*C. koseri* resistant to vancomycin. Possible aspiration.	*C. koseri* VAP	3^a^
	58		85.6	*C. glabrata* 10^3^						
23	M	Respiratory failure	NP	COF 10^4^	*S. mitis/oralis* 10^4^	COF few	Amoxi/clav acid −18- -2	Admitted with bilateral pneumonia. *M. pneumoniae* on day 7 (ct 23. IgG +)	*M. pneumoniae* CAP	11
	25		NP		*S. haemolyticus* 10^3^		Ciprofloxacin −8- -2			
					*N. subflava* 6*10^2^		Azitromycin 0-4			

^a^deceased

### Clinical parameters

**Fig. 2:**
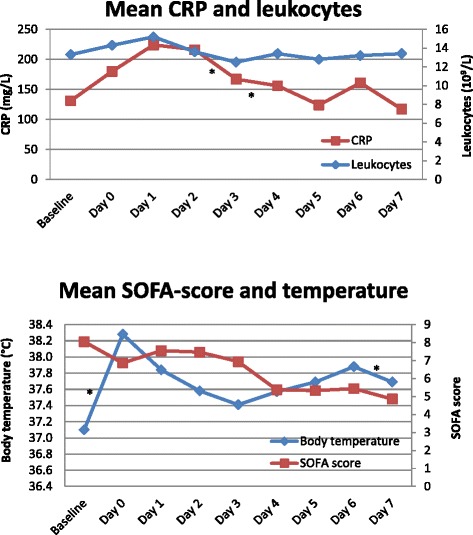
Course of clinical and laboratory parameters. Numbers signify mean values in all commensal oropharyngeal flora caused ventilator-associated pneumonia suspected cases. *P value < .05 for the increase/decrease of the value as compared to the previous value

On the day of BAL, mean CPIS revealed 6.4 (standard deviation 2.4). An increase in mean SOFA score, white blood cell count, and CRP was observed on the day after the diagnosis compared to the day of diagnosis (See Fig.  2). After this initial increase, a gradual and persistent decrease was observed for all parameters.

### Outcome

As shown in Table 1, median hospital LOS and ICU LOS in the studied group were significantly longer compared to the reference population (64 days *vs.* 24 days [*P* = .001] and 33 *vs.* 5.7 days [*P* < .001], respectively). The SMR in the studied group revealed 1.26 compared to 0.77 in the reference population (*P* = .137).

### Microbiological results

Overall cytological BAL findings are presented in Table 1 and an individual overview of cytological and microbiological analysis is provided in Table 2. In 21 patients, a BALF cell count was performed revealing 13 BALF with more than 50 % polymorphonuclear neutrophils. In 11 BALF, 2 % or more BALF cells contained ICOs. Additionally, the percentage of squamous epithelial cells, an indicator of oropharyngeal contamination, was less than 1 % in all patients. In 6 patients, BALF could not be retrieved from storage and in 3 patients, BALF culture yielded no growth. These 3 fluid samples were preserved for 5, 6, and 9 years. In the remaining 14 BALF, MALDI-TOF-MS was able to identify 1 or more bacterial species. In 5 patients, bacteria ≥ 10^4^ cfu/ml were identified, of which 2 types are potential pathogenic pathogens (*Citrobacter koseri* 10^6^ cfu/ml and *H. influenzae* 10^6^ cfu/ml) and 1 type possibly pathogenic (*E. faecalis* 2*10^4^ cfu/ml). In 4 BALF, the presence of exclusively COF according to the definition was confirmed by MALTI-TOF-MS.

Endotracheal aspirates were obtained from all studied cases. Rare, moderate and heavy growth of COF was present in 9, 1, and 4 cases, respectively. Moderate or heavy growth of COF was present in 2 out of 15 patients with probably COF-VAP [sensitivity 13 %]). In 6 cases, moderate or heavy growth of another pathogenic microorganism was identified (*S. aureus* [twice], *P. aeruginosa* [twice], *H. influenzae* [case described above in this section], and *M. morganii*). Three cases were eventually diagnosed with pneumonia caused by the same microorganism (positive predictive value 50 %). PCR on BALF for viral pathogens was performed in 15 patients. Parainfluenza virus-3 (cycle threshold (ct) 19 [infection likely]) and Cytomegalovirus (ct 37 [probably reactivation]) were identified, each virus in 1 case. In 1 patient, *Mycoplasma pneumoniae* (ct 23 [infection likely]) was identified by PCR. In 1 case, both BALF Grocott’s staining and PCR were positive for *Pneumocystis jirovecii*.

### Antibiotic treatment

Data concerning the administered antibiotics were available in 22 patients. Eleven of these patients (50 %) received (broad spectrum) antibiotics in the 72 h prior to the BAL. Overall, piperacillin/tazobactam was the most frequently used antibiotic (15/22 [68 %]). Five patients received inadequate treatment for COF-VAP (See Table 2; Patients no. 7, 8, 10, 22, and 23) of which 2 had unfavourable respiratory outcome (patients no. 8 and 10) and 2 died (patients no. 10 and 22), 47 and 3 days after the diagnosis, respectively. Of the 17 patients that received appropriate COF-VAP treatment, 6 patients died after a median of 54 days after the diagnosis.

### Post mortem examination

In 4 out of the 9 deceased patients, consent for autopsy was obtained. Pathological evidence of an active pneumonia was found in 3 patients in post-mortem examination, performed at least 22 days after diagnosis. A faecal peritonitis was observed in the fourth patient, probably explaining her respiratory deterioration.

### Level of certainty of the presence of commensal oropharyngeal flora ventilator-associated pneumonia

An alternative diagnosis was concerned more likely in 6 patients: *Citrobacter koseri* VAP, *H. influenzae* pneumonia, parainfluenza virus-3 CAP or *E. faecium* HAP, *Mycoplasma pneumoniae* CAP (all 4 with clinical signs of pneumonia), *Pneumocystis jirovecii* caused CAP (with CT-thorax suggestive of *Pneumocystis* pneumonia), and abdominal sepsis (with clinical signs of an ileus the day before the patient deceased). Eleven patients received antibiotics in the three days prior to the (presumed) VAP, making their culture results more difficult to interpret. The cultures in cases number 2 and 18 yielded ≥10^3^ cfu/ml *P. aeruginosa* and *S. aureus*, respectively, under appropriate antibiotic treatment, which cannot exclude their causative contribution to VAP. Therefore these 2 cases were considered to have possible COF-VAP. In the remaining 15 cases (9.4 % of all VAP cases), no other infection was found and COF-VAP was considered likely.

### Additional findings

Additionally, 3 cases of probable COF-HAP and 1 case of probable COF-HAP were identified in 8 eligible cases (see Appendix). Besides the fact that these 4 positive cases were immunocompromised due to previous used medication or malignancies, further discussion of this subgroup is beyond the scope of this article.

## Discussion

This large retrospective analysis of a prospectively collected case series revealed 23 patients suspected of COF-VAP. In 15 patients (9.4 % of all VAP cases), COF-VAP was the most likely diagnosis.

### Previous studies

So far, 1 currently available study investigated the possible role of COF as the cause of VAP [39]. This French group retrospectively investigated 29 episodes of VAP with significant growth of oropharyngeal and cutaneous commensal microorganisms in BALF and protected brush specimen derived from 28 immunocompetent patients. The suspected cases also revealed signs of VAP including a longer ICU LOS. A panel of 3 experts confirmed 14 out of the 29 (48 %) suspected cases as COF-VAP (corresponding to 3.8 % of their VAP cases), whereas in the other cases an alternative diagnosis was considered more likely. The authors concluded that COF may cause VAP in ICU patients with no known prior immunodeficiency, that it has similar clinical features as VAP, and that patients should be treated with antibiotics with the probable exception of stable patients with a high likelihood of an alternative diagnosis [39]. The results of the present study, using more clinical data and microbiological analyses, are in support of their preliminary findings.

Two decades ago, several small studies revealed the presence of COF in post-mortem lung biopsy cultures in previously mechanically ventilated patients [40–42] (reported incidences varied from 9 % to 57 %, with not all studies reporting the level of growth), whereas another study found no COF in post-mortem biopsy diagnosed VAP cases [43].

### Epidemiology

One might perhaps expect a rise in COF-VAP rates after the introduction of SOD/SDD, as it focusses on the elimination of Gram negatives as well as the selection of commensal bacteria [44]. Yet, so far (see Table 2) no diagnosis of COF-VAP was made after the introduction of SOD/SDD, whereas the incidence of VAP per 1,000 ventilator days declined from 4.38 ± 1.64 before to 1.64 ± 0.43 (*P* = .007) after the introduction of SOD and SDD in our clinic [45].

### Results of endotracheal aspirates

Whereas ETA and BALF analysis can both be used to diagnose VAP [14, 30], results of these 2 diagnostic modalities agree only fairly [46]. Overall, the current results confirm the poor agreement between BALF and ETA analyses results.

### Re-cultured bronchoalveolar lavage fluid and identification with matrix-assisted laser desorption/ionization time-of-flight mass spectrometry

Whereas all 23 BALF samples revealed ≥ 10^4^ cfu/ml COF at the time of the BAL procedure, the repeated microbiological examinations on preserved specimen were frequently not in accordance. The BALF samples that revealed no growth were similar durations as the samples that did reveal growth, suggesting that the time of storage did not cause the disagreement. However, *Neisseria* spp., *Streptococcus* spp. (not *S. pyogenes*), and *Haemophilus* spp. are known with a limited survival on inanimate surfaces [47], contributing to the absence of growth on re-culturing despite the correct solvents and temperature. The lack of significant growth of microorganism could have been related to the storage-related factors, as well as the presence of antibiotics in the sample before storage. On the other hand, one may doubt if the *C. koserii, H. influenzae,* and *E. faecalis* identified by MALDI-TOF-MS were true pathogens or that their presence was caused by contamination before, during, or after the BALF storage and retrieval.

Matrix-assisted laser desorption/ionization time-of-flight mass spectrometry was able to identify all strains that re-grew on any agar plate. *Rothia dentocariosa*, *Capnocytophaga sputigena*, and *Lactobacillus acidophilus* are part of COF, although not included in our definition since these species were not routinely identified before MALDI-TOF-MS use.

### Diagnosis

Although some results are more difficult to interpret due to previous antibiotic use, identified COF is mostly susceptible to the antibiotics used, but was nonetheless still present in significant numbers in BALF. Furthermore, at least 9 patients that were eventually diagnosed with COF-VAP did not receive antibiotics prior to BAL. In 6 patients, an alternative diagnosis was concerned more likely, although COF as a contributing or the main cause of VAP can neither be established nor excluded with certainty. Remarkably, 3 suspected VAP cases actually had CAP.

### Treatment and outcome

Four out of the 15 patients with probable COF-VAP received inappropriate antibiotic treatment. The respiratory outcome was unfavourable in 2 out of 3 cases that survived the VAP episode, suggesting that COF-VAP should be treated appropriately. Commensal oropharyngeal flora is generally susceptible to the antibiotics suggested by authoritative guidelines [14].

### Entity or fiction?

Although no hard argument can be provided to state that COF-VAP is an entity, 6 arguments originated from the present study support that COF indeed may cause VAP. First, it is plausible from a pathophysiological point of view. As stated in the introduction section, COF may behave pathogenic in immunocompromised patients. Whereas COF-VAP developed after a median of 8 days of mechanical ventilation, a previously immunocompetent person may already be considered immunocompromised after 48 h of ICU admission [24]. Additionally, many COF-VAP suspected cases revealed positive viral and fungal PCR, which is also associated with a decreased immune status. Finally, COF was able to cause HAP and CAP in immunocompromised patients. Second, scores resulting from generally accepted clinical scoring systems, as well as laboratory results, indicated a significant clinical relevance. Third, cytological analysis of BALF frequently was indicative of a bacterial infection. Fourth, COF was the most likely cause of VAP in 15 cases and possibly the cause in 2 cases. Fifth, inappropriate antibiotic treatment for COF-VAP (n = 4) was associated with unfavourable respiratory outcome. Sixth and last, COF-VAP was associated with increased ICU and hospital LOS including a trend towards increased mortality as compared to a reference population.

Contrariwise, post-mortem examination 4 days after BAL of one patient revealed no signs of pneumonia, indicative of a false positive BALF analysis. However, at the time of BAL, this patient presents with an ileus and abdominal sepsis was the post-mortem diagnosis. Although it is possible that the patient fulfilled all criteria for performing a BAL due to the extrapulmonary problems, BAL should thus only be performed in the absence of an obvious alternative explanation for the patient’s clinical presentation.

The current study demonstrated a probable association between merely significant growth of COF in VAP suspected patients and worse outcome. Similar to VAP caused by other microorganisms [2], it remains unknown whether this association is causal or that COF-VAP results from critical illness and that the outcome is therefore unfavourable.

### Limitations of the study

There are several limitations to the study. First, due to the lack of a globally accepted gold standard to diagnose VAP [7, 8, 11], VAP in clinically suspected patients was defined as BALF revealing ≥ 2 % ICOs and/or significant growth (≥ 10^4^ cfu/ml [36]) of a potential pathogenic microorganism. Second, a limited number of clinical and microbiological data were irretrievable in a number of patients. Third, median ICU and hospital LOS of the suspected cases were compared to mean LOS in a recent patient population. Since mean ICU and hospital LOS declined during the studied period (2005–2013), this comparison may be unfair. However, since the mean ICU LOS was 8.6 days in 2005, a median ICU LOS of 33 days in the studied period is still significantly higher. Fourth, enterococci were excluded as a potential cause for VAP, although these microorganisms may possibly also cause VAP in the immunocompromised ICU-patient. Fifth, a reference population is not a control group. In future studies, a control group should preferably consist of “regular” VAP cases to demonstrate that COF behaves like “regular” VAP causative microorganisms. Finally, in the process of BALF storage and re-culturing the occurrence of contamination cannot be ruled out.

### Future studies

Since COF-VAP has a low overall incidence (15 out of 6500 ICU admissions [0.23 %]) and VAP incidences decline [45, 48], future research should preferably focus on multi-centre trials. Notwithstanding, when more similar studies become available, a meta-analysis could empower the results of Lambotte *et al.* and the current study.

## Conclusions

As COF was identified as the most likely causative agent in 9.4 % of all VAP episodes, COF is probably an overlooked cause of VAP. The immunocompromised status of the ICU patient may contribute to its origination. Commensal oropharyngeal flora VAP is probably associated with significant clinical signs of bacterial infection, a prolonged ICU and hospital LOS, and a trend towards increased mortality. In the absence of other pulmonary and non-pulmonary explanations for a patient’s pulmonary deterioration, ICU physicians should perhaps acknowledge this entity and treat it accordingly.

## Appendix

**Table 3:** Individual characteristics, diagnostic results and outcome of the suspected cases of commensal oropharyngeal flora caused hospital-acquired pneumonia and community-acquired pneumonia

Case	Sex	Admission indication	BAL fluid analysis			Endotracheal aspirate Semiquantitativly	Antibiotic treatment (days [0 = diagnose])	Additional characteristics	Diagnosis	ICU
	Age		% ICO	BAL fluid culture	MALDI-TOF-MS and re-culturing					LOS
			% PMN	cfu/ml	(cfu/ml)					
Suspected cases of commensal oropharyngeal flora caused hospital-acquired pneumonia										
24	M	Respiratory failure	37.4	COF 10^5^	Specimen storage lacking	COF heavy	Piper/tazob −6 -1	Multiple myeloma. Post-mortem exam: CMV pneumonia. *Candida* spp.	CMV pneumonia	1^a^
	65		33.0	*C. glabrata* 10^3^			Metronidazole −11 -1			
							Fluconazole −11-1			
25	M	Respiratory failure	0.0	COF 10^5^	No growth	E. coli moderate	None	Immunosuppresive drug after lung transplantation. Ventilator depended after discharge	COF-HAP	182
	41		60.0			OF rare				
26	F	Neurological	4.8	COF > 10^5^	*N. subflava* 4*10^2^	COF few	Amoxi/clav acid −2 -5	Possible aspiration Post-mortem exam: Indicative for amiodarone induced pneumonitis (AIP)	AIP	10^a^
	73		63.8		*S. anginosus* 3*10^2^					
					*S. mitis/oralis* 3*10^2^					
					*H. influenzae* 2*10^2^					
27	M	Hypokalemia	0.0	COF 10^5^	*E. faecium* 4*10^4^	No growth	Ciprofloxacin −2 -2	Acute myeloid leukemia. RSV ct 20, HSV-1 ct 29	RSV CAP/ *E. faecium* HAP	29^a^
	60		96.8		*S. epidermidis* 3*10^4^		Piper/tazob −2-18			
							Vancomycin 1-26			
28	M	Respiratory failure	0.0	COF 10^4^	*C. jeikeium* 5*10^3^	Candida spp.	Piper/tazob −11 -0	B-cel Lymphoma	COF-HAP	16^a^
	50		11		*E. faecium* 4*10^3^		Meropenem 0-16			
29	M	Respiratory failure	0.0	COF 10^5^	*E. faecium* 2*10^4^	COF few	Piper/tazob −2 -15	Acute myeloid leukemia	COF-HAP	31^a^
	54		0.8				Vancomycin 4-12	Possible aspiration.		
								HSV-1 ct 34		
Suspected cases of commensal oropharyngeal flora caused community-acquired pneumonia										
30	F	Sepsis	0.0	COF 10^4^	No growth	NP	Amoxi/clavc acid 0-1	Metastatic mammacarcinoma	COF-CAP	1^a^
	48		21.4				Ciprofloxacin 0-1			
31	M	Respiratory failure	0.2	COF 10^4^	*A. viscosus*	COF rare	Co-trimoxazole −20 -27	Idiopathic CD4 deficiency. ICU admission for BAL only. *P. jiroveci* ct 24.	PcP	0
	68		32.8		*R. mucilaginosa*					
					*S. mutans*					

^a^deceased

Abbreviations, not used in the main text: HSV: Herpes simplex virus; RSV: respiratory syncytial virus

## Abbreviations

ADV: Adenovirus
APACHE: Acute physiology and chronic health evaluation
BAL: Bbronchoalveolar lavage
BAL: Bronchoalveolar lavage fluid
CAP: Community-acquired pneumonia
CLL: Chronic lymphocytic leukaemia
CMV: Cytomegalovirus
CNS: Coagulase-negative staphylococcus
CPAP: Continues positive airway pressure
CPIS: Clinical pulmonary infection score
CRP: C-reactive protein
ct: Cycle threshold
ETA: Endotracheal aspirate
F: Female
HAP: Hospital-acquired pneumonia
ICO: Intracellular organism, in Table, percentage of BAL fluid cells containing intracellular organisms
ICU: Intensive care unit
IQR: Interquartile range
LOS: Length of stay
M: Male
MALDI-TOF-MS: Matrix-assisted laser desorption/ionization time-of-flight mass spectrometry
MV: Mechanical ventilation
NP: Not performed
OSAS: Obstructive sleep apnoea syndrome
PcP: *Pneumocystis* pneumonia
PCR: Polymerase chain reaction
PIV: Parainfluenza virus
PMN: Polymorphonuclear neutrophils
SD: Standard deviation
SDD: Selective digestive tract decontamination
SMR: Standardized mortality ratio
SOD: Selective oropharyngeal decontamination
SOFA: Sequential organ failure assessment
VAP: Ventilator-associated pneumonia
