# Erratum to: ‘Ventilator-associated Pneumonia caused by commensal oropharyngeal a retrospective Analysis of a prospectively collected Database’

**DOI:** 10.1186/s12890-015-0098-8

**Published:** 2015-09-17

**Authors:** Johannes B. J. Scholte, Johan I. M. van der Velde, Catharina F. M. Linssen, Helke A. van Dessel, Dennis C. J. J. Bergmans, Paul H. M. Savelkoul, Paul M. H. J. Roekaerts, Walther N. K. A. van Mook

**Affiliations:** Department of Intensive Care Medicine, Luzerner Kantonspital, Luzern 16, 6000 Switzerland; Department of Intensive Care Medicine, Maastricht University Medical Centre+, P.O. box 5800, Maastricht, 6202 AZ The Netherlands; Department of Medical Microbiology, Atrium Medical Centre, P.O. box 4446, Heerlen, 6401 CX The Netherlands; Department of Medical Microbiology, Maastricht University Medical Centre+, P.O. box 5800, Maastricht, 6202 AZ The Netherlands

## Erratum

Unfortunately, the original version of this article [1] contained an error. The title was stated incorrectly. The correct title: “Ventilator-associated Pneumonia caused by commensal oropharyngeal Flora; a retrospective Analysis of a prospectively collected Database” has been corrected in the original article and is also included correctly below.
